# Reduced p21^WAF1/CIP1^protein expression is predominantly related to altered p53 in hepatocellular carcinomas

**DOI:** 10.1054/bjoc.2000.1310

**Published:** 2000-06-02

**Authors:** Y-Z Shi, A-M Hui, T Takayama, X Li, X Cui, M Makuuchi

**Affiliations:** Hepato-Biliary-Pancreatic Surgery Division, Department of Surgery, Graduate School of Medicine, University of Tokyo, 7-3-1 Hongo, Tokyo, Bunkyo0ku, 113-0033, Japan

**Keywords:** p21^WAF1/CIP1^, p53, hepatocellular carcinoma

## Abstract

To investigate the relationship between the expression of p21^WAF1/ClP1^protein and p53 status and the possible role of the two proteins in hepatocellular carcinomas (HCCs), we examined the expression of p21^WAF1/CIP1^and p53 immunohistochemically in 81 tumours from 65 patients with hepatocellular carcinoma. p21^WAF1/CIP1^protein was absent from 59 of 81 tumours (72.8%), and altered p53 expression was found in 43 (53.1%). p21^WAF1/CIP1^expression was significantly associated with p53 status (*P*= 0.0008); 38 of 59 tumours lacking p21^WAF1/CIP1^protein were accompanied by altered p53 expression. Further analyses showed that p21^WAF1/CIP1^expression was inversely correlated with p53 expression in hepatitis C virus (HCV)-related HCCs, but not in HBV-related hepatocellular carcinomas and hepatocellular carcinomas without viral infection. All 11 tumours with intrahepatic metastasis showed altered p21^WAF1/CIP1^or p53 expression. In contrast, no intrahepatic metastasis was found in any of the 17 tumours without abnormal expression of either of the two proteins. These results suggest that: (1) different modes of p21^WAF1/CIP1^regulation are involved in HCCs differing in their hepatitis viral infection status, and p21^WAF1/CIP1^expression appears to be predominantly related to altered p53 in HCV-related HCCs; (2) disruption of the p53–p21^WAF1/CIP1^cell- cycle-regulating pathway may contribute to malignant progression of HCC. © 2000 Cancer Research Campaign


*British Journal of Cancer* (2000) **83**(1), 50–55
© 2000 Cancer Research Campaign
doi: 10.1054/ bjoc.2000.1310, available online at http://www.idealibrary.com on IDEAL®

### Reduced p21$^{WAF1/CIP1}$ protein expression is predominantly related to altered p53 in hepatocellular carcinomas

**Y-Z Shi, A-M Hui, T Takayama, X Li, X Cui and M Makuuchi**

Hepato-Biliary-Pancreatic Surgery Division, Department of Surgery, Graduate School of Medicine, University of Tokyo, 7-3-1 Hongo, Bunkyo0ku, Tokyo 113-0033, Japan

**Summary** To investigate the relationship between the expression of p21$^{WAF1/CIP1}$ protein and p53 status and the possible role of the two proteins in hepatocellular carcinomas (HCCs), we examined the expression of p21$^{WAF1/CIP1}$ and p53 immunohistochemically in 81 tumours from 65 patients with hepatocellular carcinoma. p21$^{WAF1/CIP1}$ protein was absent from 59 of 81 tumours (72.8%), and altered p53 expression was found in 43 (53.1%). p21$^{WAF1/CIP1}$ expression was significantly associated with p53 status ($P$ = 0.0008); 38 of 59 tumours lacking p21$^{WAF1/CIP1}$ protein were accompanied by altered p53 expression. Further analyses showed that p21$^{WAF1/CIP1}$ expression was inversely correlated with p53 expression in hepatitis C virus (HCV)-related HCCs, but not in HBV-related hepatocellular carcinomas and hepatocellular carcinomas without viral infection. All 11 tumours with intrahepatic metastasis showed altered p21$^{WAF1/CIP1}$ or p53 expression. In contrast, no intrahepatic metastasis was found in any of the 17 tumours without abnormal expression of either of the two proteins. These results suggest that: (1) different modes of p21$^{WAF1/CIP1}$ regulation are involved in HCCs differing in their hepatitis viral infection status, and p21$^{WAF1/CIP1}$ expression appears to be predominantly related to altered p53 in HCV-related HCCs; (2) disruption of the p53–p21$^{WAF1/CIP1}$ cell-cycle-regulating pathway may contribute to malignant progression of HCC. © 2000 Cancer Research Campaign

**Keywords:** p21$^{WAF1/CIP1}$; p53; hepatocellular carcinoma

Cell cycle progression is governed by several checkpoints, which are regulated by a family of protein kinases, the cyclin-dependent kinases (CDKs) and the cyclins (Hunter and Pines, 1994). The restriction point is one of the most important checkpoints in the late G1 phase. Disruption of the checkpoints is one mechanism of oncogenesis. p21$^{WAF1/CIP1}$ protein, a universal CDK inhibitor (Xiong et al, 1993), and p53 protein are two important components of the G1 restriction point.

Recently, the p21$^{WAF1/CIP1}$ gene was cloned and mapped to the 6p21.2 chromosome region (El-Deiry et al, 1993; Noda et al, 1994). p21$^{WAF1/CIP1}$ inhibits a wide variety of cyclin–CDK complex activities by binding to the complexes (Harper et al, 1993; Xiong et al, 1993). p21$^{WAF1/CIP1}$ has also recently been shown to bind to and inactivate proliferation cell nuclear antigen, the processivity subunit of DNA polymerase δ (Waga et al, 1994). These observations suggest that p21$^{WAF1/CIP1}$ may play a dual role in blocking entry into S phase (Noda et al, 1994). In addition, p21$^{WAF1/CIP1}$ has also been suggested to play a role in inducing differentiation and apoptosis (Michieli et al, 1994; Sheikh et al, 1995), and introduction of p21$^{WAF1/CIP1}$ cDNA suppresses the growth of human tumour cells in culture (El-Deiry et al, 1993).

Cells lacking functional p53 express a very low level of p21$^{WAF1/CIP1}$ and the p21$^{WAF1/CIP1}$ promoter contains a p53-binding site, suggesting that expression of p21$^{WAF1/CIP1}$ depends on p53 function (El-Deiry et al, 1993, 1994; Xiong et al, 1993). However, p21$^{WAF1/CIP1}$ can be inducible in p53-null cells, showing that the expression of p21$^{WAF1/CIP1}$ can also be induced by p53-independent pathways (Michieli et al, 1994; Zhang et al, 1995).

We previously investigated p21$^{WAF1/CIP1}$ mRNA expression by reverse-transcriptase polymerase chain reaction (RT-PCR) and p53 mutational status by PCR single-strand conformation polymorphism (SSCP) and direct DNA sequencing in hepatocellular carcinomas (HCCs). We suggested that p21$^{WAF1/CIP1}$ mRNA expression is regulated predominantly by a p53-dependent pathway and that reduced p21$^{WAF1/CIP1}$ mRNA expression may contribute to hepatocarcinogenesis (Hui et al, 1997). However, we did not evaluate the expression of p21$^{WAF1/CIP1}$ and p53 at the protein level. Because post-transcriptional regulation is also an important mechanism in gene expression (Hui et al, 1996*b*; Loda et al, 1997; Maki and Howley, 1997), and loss of function of p53 is caused not only by gene mutation but also when p53 protein interacts with viral or cellular oncoproteins (Sarnow et al, 1982; Farmer et al, 1992; Yew and Berk, 1992; Steegenga et al, 1996; Somasundaram and El-Deiry, 1997), it is necessary to investigate p21$^{WAF1/CIP1}$ and p53 at the protein level. The wild-type p53 protein has a short half-life of about 20 min, and in general it cannot be detected by immunohistochemistry. However, a p53 gene mutation or p53 interacting with oncoproteins may stabilize p53 protein and result in altered expression of p53 that can be detected by immunohistochemistry (Sarnow et al, 1982; Finlay et al, 1988; Iggo et al, 1990; Hall et al, 1991; Farmer et al, 1992; Yew and Berk, 1992; Steegenga et al, 1996; Somasundaram and El-Deiry, 1997).

We investigated the expression of p21$^{WAF1/CIP1}$ and p53 proteins in 81 tumours from 65 patients with HCC by immunostaining to determine whether p21$^{WAF1/CIP1}$ expression depends on p53 functional status, and the relationship between the two proteins' expression and clinicopathological features.

*Received 14 July 1999*
*Revised 14 February 2000*
*Accepted 24 February 2000*

*Correspondence to:* A-M Hui

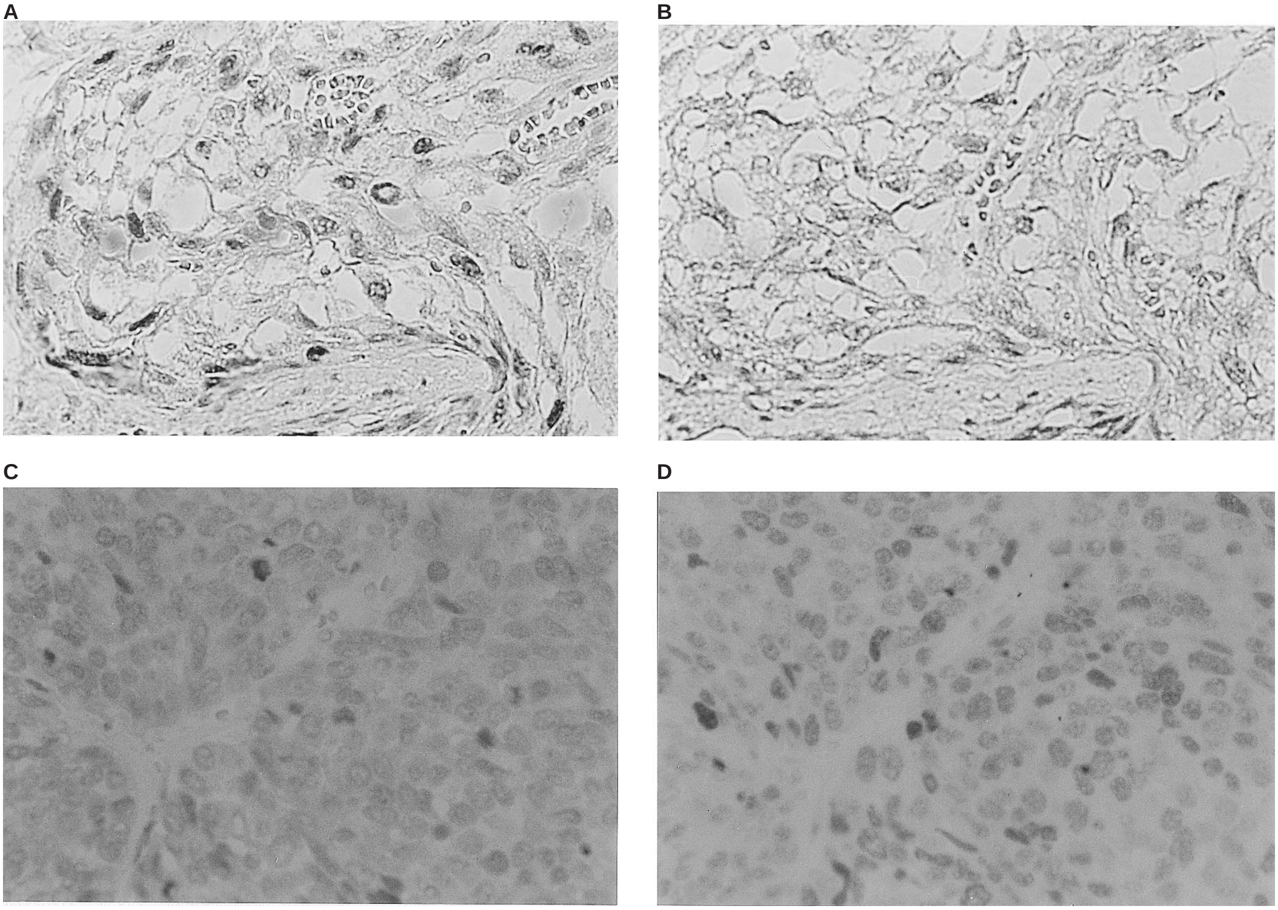

**Figure 1** Representative examples of immunohistochemical staining of $p21^{WAF1/CIP1}$ and p53. Tumour shows positive nuclear staining for $p21^{WAF1/CIP1}$ (**A**) while not for p53 (**B**). Tumour is completely negative for $p21^{WAF1/CIP1}$ (**C**), while p53 is diffusely stained (**D**). (× 350)

### MATERIALS AND METHODS

### Patients and specimens

Eighty-one primary HCC tissues were obtained from 65 patients who underwent surgical resection at the Hepato-Biliary-Pancreatic Surgery Division, Department of Surgery, Graduate School of Medicine, University of Tokyo, Tokyo, Japan. Fifty patients were found to have underlying cirrhosis, and 15 had chronic hepatitis. The study included 13 (20%) women and 52 (80%) men with a mean age of 61 years (range 13–74 years). Fifty-six patients had serological evidence of viral infection (11 were positive for hepatitis B surface antigen [HBsAg], 43 were positive for hepatitis C virus [HCV] antibody and two were positive for both markers). The remaining nine patients were negative for both hepatitis markers. Tissue samples were obtained at the time of operation, fixed in 10% formalin, and histopathologically examined. The size of the tumours varied from 0.6 to 15 cm (mean 4.0 ± 3.5 s.d.). The 81 tumours comprised 23 well-, 43 moderately, and 15 poorly differentiated HCCs. Portal vein tumour thrombi were found in 18 (22.2%) HCCs, and intrahepatic metastatic lesions were found in 11 (13.6%).

### Immunohistochemical staining for $p21^{WAF1/CIP1}$ and p53

Immunohistochemistry was done using the avidin–biotin–peroxidase complex method. Paraffin-embedded sections (4 μm-thick) were deparaffinized in xylene, rehydrated in decreasing concentrations of ethanol and then treated with 3% hydrogen peroxide in methanol for 30 min to block endogenous peroxidase activity. After brief washing with distilled water, tissue sections were processed in 10 mM citrate buffer (pH 6.0) and heated to 120°C in an autoclave for 10 min for antigen retrieval (Hui et al, 1999*a*, 1999*b*, 2000; Li et al, 2000). Slides were allowed to cool at room temperature for 20 min and then rinsed with phosphate-buffered saline (PBS). To inhibit non-specific binding activity, slides were incubated with blocking serum at room temperature for 30 min. Sections then were incubated with primary monoclonal antibody against $p21^{WAF1/CIP1}$ (clone EA10, Oncogene Science, Cambridge, MA, USA) diluted at 1:200, or with monoclonal antibody against p53 (clone DO-7; Dako A/S, Denmark) at 1:100, at 4°C in a moist chamber overnight. The sections were then incubated with biotinylated anti-mouse immunoglobulins (Vector Laboratories, Inc., Burlingame, CA, USA) for 30 min, and avidin–biotin complex (Vector Laboratories) for 30 min at room temperature, with washing in PBS before each incubation. 3,3′-Diaminobenzidine tetrahydrochloride was used as the colour reagent, and haematoxylin was used as a counterstain. Normal oesophageal squamous epithelium and HCC with known p53 gene mutation and p53 protein overexpression were used as positive controls for $p21^{WAF1/CIP1}$ and p53 respectively. Negative controls were obtained by omitting primary antibody. Only nuclear staining was considered to be positive for $p21^{WAF1/CIP1}$ and p53. Based on

© 2000 Cancer Research Campaign

**Table 1** Relationship between p21$^{WAF1/CIP1}$ and p53 protein expression and clinicopathological features in HCCs

| Clinicopathology | p21$^{WAF1/CIP1}$ (–) | *P*-value | p53 (+) | *P*-value |
|---|---|---|---|---|
| Virus infection[a] | | | | |
| HBV | 11/13 (84.6%) | 0.39 | 5/13 (38.5%) | 0.71 |
| HCV | 37/54 (68.5%) | | 30/54 (55.6%) | |
| HBV + HCV | 3/5 (60.0%) | | 3/5 (60.0%) | |
| Negative | 8/9 (88.9%) | | 5/9 (55.6%) | |
| Background liver disease | | | | |
| Chronic hepatitis | 15/18 (83.3%) | 0.46 | 9/18 (50.0%) | 0.85 |
| Liver cirrhosis | 44/63 (69.8%) | | 34/63 (54.0%) | |
| Tumour size (cm) | | | | |
| <2 | 21/29 (72.4%) | 0.52 | 15/29 (51.7%) | 0.73 |
| 2–5 | 23/29 (79.3%) | | 17/29 (58.6%) | |
| ≥5 | 15/23 (65.2%) | | 11/23 (47.8%) | |
| Tumour differentiation | | | | |
| Well | 15/23 (65.2%) | 0.40 | 8/23 (34.8%) | * |
| Moderate | 34/43 (79.1%) | | 23/43 (53.5%) | ** |
| Poor | 10/15 (66.7%) | | 12/15 (80.0%) | |
| Intrahepatic metastasis | | | | |
| Positive | 9/11 (81.8%) | 0.47 | 7/11 (63.6%) | 0.45 |
| Negative | 50/70 (71.4%) | | 36/70 (51.4%) | |
| Portal involvement | | | | |
| Positive | 12/18 (66.7%) | 0.50 | 13/18 (72.2%) | 0.06 |
| Negative | 47/63 (74.6%) | | 30/63 (47.6%) | |
| Venous involvement | | | | |
| Positive | 4/7 (57.1%) | 0.33 | 4/7 (57.1%) | 0.82 |
| Negative | 55/74 (74.3%) | | 39/74 (52.7%) | |

[a] HBV, HBsAg-positive; HCV, anti-HCV-antibody-positive; HBV + HCV, both markers positive; Negative, both markers negative. *Well vs poorly differentiated, $P = 0.0064$; **Moderately vs poorly differentiated, $P = 0.07$.

the previously published criteria, positive staining of p21$^{WAF1/CIP1}$ was considered when ≥ 5% of tumour cells were stained (Ogawa et al, 1997). Positive scoring for p53 was considered when ≥ 10% of the nuclei were stained, according to the criteria used previously (Esrig et al, 1994; Cote et al, 1998).

### Statistical analysis

$\chi^2$ test or Fisher's exact test was used to examine differences and relationships between groups of patients classified by p21$^{WAF1/CIP1}$ and p53 staining. Differences at $P < 0.05$ were judged to be statistically significant.

### RESULTS

### p21$^{WAF1/CIP1}$ expression p21$^{WAF1/CIP1}$ immunoreactivity was always nuclear, and no cytoplasmic staining of p21$^{WAF1/CIP1}$ was seen in any specimen. The HCCs showed heterogeneous expression of p21$^{WAF1/CIP1}$; the percentage of p21$^{WAF1/CIP1}$-positive cells ranged from 0% to 60% (median 5%). Twenty-two of 81 HCCs (27.2%) showed positive staining for p21$^{WAF1/CIP1}$ protein (Figure 1A). Most (86.4%) p21$^{WAF1/CIP1}$-negative tumours showed < 1% positive cells or complete loss of immunoreactivity. p21$^{WAF1/CIP1}$ expression was not associated with viral infection, histological features of non-tumourous livers, tumour size, tumour differentiation, intrahepatic metastasis, or tumour portal or hepatic vein involvement ($P > 0.05$; Table 1).

### p53 expression

Forty-three of 81 HCCs (53.1%) showed positive staining for p53 protein (Figure 1D). No positive p53 expression was observed in the corresponding non-tumourous liver tissue. p53 overexpression was more frequent in moderately differentiated tumours (23/43 HCCs, 53.5%) than in well-differentiated tumours (8/23 HCCs, 34.8%), and was even more frequent in poorly differentiated tumours (12/15 HCCs, 80%) (poorly vs well-differentiated, $P = 0.0064$; Table 1). A trend approaching significance was observed between positive p53 expression and tumour portal vein invasion ($P = 0.06$; Table 1). No association was found between positive p53 expression and any other clinicopathological parameters examined.

### Relationship between p21$^{WAF1/CIP1}$ and p53 expression

The expression of p21$^{WAF1/CIP1}$ was significantly associated with p53 status ($P = 0.0008$, Table 2); 38 of the 43 tumours (88.4%) with aberrant p53 expression showed loss of p21$^{WAF1/CIP1}$ expression and 38 of the 59 tumours (64.4%) without p21$^{WAF1/CIP1}$ expression were accompanied by altered p53 expression (two tumours showing an inverse staining pattern of p21$^{WAF1/CIP1}$ and p53 are shown in Figure 1 A–D). However, 21 of the 59 (35.6%) tumours lacking p21$^{WAF1/CIP1}$ protein showed p53-negative staining, and five tumours showed concurrent positive staining of p21$^{WAF1/CIP1}$ and p53.

The relationship between p21$^{WAF1/CIP1}$ and p53 expression was further evaluated in HCV-related HCCs, HBV-related HCCs and HCCs with no virus infection (Table 2). The expression of

*© 2000 Cancer Research Campaign*

**Table 2** Relationship between $p21^{WAF1/CIP1}$ and p53 expression in HCCs

| | No. | $p21^{WAF1/CIP1}$-positive | $p21^{WAF1/CIP1}$-negative | *P*-value |
|---|---|---|---|---|
| All HCCs | | | | |
| p53-positive | 43 | 5/43 (11.6%) | 38/43 (88.4%) | |
| p53-negative | 38 | 17/38 (44.7%) | 21/38 (55.3%) | 0.0008 |
| No. | 81 | 22 | 59 | |
| HCV-related HCCs | | | | |
| p53-positive | 30 | 4/30 (13.3%) | 26/30 (86.7%) | |
| p53-negative | 24 | 13/24 (54.2%) | 11/24 (45.8%) | 0.0013 |
| No. | 54 | 17 | 37 | |
| HBV-related HCCs | | | | |
| p53-positive | 5 | 1/5 (20.0%) | 4/5 (80.0%) | |
| p53-negative | 8 | 1/8 (12.5%) | 7/8 (87.5%) | 0.72 |
| No. | 13 | 2 | 11 | |
| HCCs with no hepatitis virus infection | | | | |
| p53-positive | 5 | 0/5 (0%) | 5/5 (100%) | |
| p53-negative | 4 | 1/4 (25.0%) | 3/4 (75.0%) | 0.24 |
| No. | 9 | 1 | 8 | |

Five HCCs positive for both HCV antibody and HBsAg were included in neither HCV-related HCCs nor HBV-related HCCs.

$p21^{WAF1/CIP1}$ was significantly correlated with p53 expression in HCV-related HCCs, but not in HBV-related HCCs or HCCs without virus infection.

The tumours were divided into two groups based on the expression patterns of the two proteins: I, $p21^{WAF1/CIP1}$+/p53– (17 HCCs; no abnormality in either protein); and II, $p21^{WAF1/CIP1}$+/p53+, $p21^{WAF1/CIP1}$–/p53– or $p21^{WAF1/CIP1}$–/p53+ (64 HCCs; at least one altered expression involved in the two proteins). When this was done, a trend approaching significance was observed between the two groups ($P = 0.066$); intrahepatic metastasis was observed more frequently in group II (11/64, 17.2%) than in group I (0/17, 0%). There was no significant relationship between either group and any of the clinicopathological features examined ($P > 0.05$).

### DISCUSSION

$p21^{WAF1/CIP1}$ plays a key role in p53-mediated arrest of the cell cycle in response to DNA damage (E1-Deiry et al, 1993, 1994; Xiong et al, 1993; Dulic et al, 1994). $p21^{WAF1/CIP1}$ inhibits transition from G1 to the S phase by inhibiting a wide variety of cyclin–CDK complexes, including cyclin D–CDK4, cyclin D–CDK2, and cyclin E–CDK2 (Gu et al, 1993; Harper et al, 1993; Xiong et al, 1993). Strikingly, in normal cells, $p21^{WAF1/CIP1}$ is associated with quaternary complexes of most cyclins, CDKs, and proliferation cell nuclear antigens, but is absent from these complexes in most transformed cells (Xiong et al, 1993). These observations suggest that reduction or loss of $p21^{WAF1/CIP1}$ expression plays an important role in tumorigenesis. Our results showed that absence of $p21^{WAF1/CIP1}$ expression was very common (59/81; 72.8%) in HCCs, suggesting that the absence of $p21^{WAF1/CIP1}$ expression may be involved in hepatocarcinogenesis.

There are several possible mechanisms that down-regulate $p21^{WAF1/CIP1}$ expression. First, the expression of $p21^{WAF1/CIP1}$ is transcriptionally induced by wild-type but not mutant p53 (El-Deiry et al, 1993). We have shown previously that HCCs with wild-type p53 express significantly greater $p21^{WAF1/CIP1}$ mRNA than tumours with mutant p53 (Hui et al, 1997). In the present study, absence of $p21^{WAF1/CIP1}$ protein was significantly associated with altered p53 expression ($P = 0.0008$). Drawing together the observations of our present and previous (Hui et al, 1997) studies, we suggest that the p53-dependent transcriptional pathway is the main mechanism that regulates $p21^{WAF1/CIP1}$ expression in HCCs.

Second, 21 of the 59 (35.6%) tumours lacking $p21^{WAF1/CIP1}$ protein showed p53-negative staining. Considering that negative immunoreactivity of p53 reflects functional p53, we hypothesize that, in these cases, $p21^{WAF1/CIP1}$ is probably down-regulated by other factor(s). Recently, it has been reported that HCV core protein suppresses the transcriptional activity of the $p21^{WAF1/CIP1}$ promoter (Ray et al, 1998). In the present study, 59 (including five HCCs positive for both HCV and HBV markers) of the 81 HCCs were associated with HCV infection. Therefore, we consider it is highly probable that HCV core protein down-regulates $p21^{WAF1/CIP1}$ expression in HCV-related HCCs. In this study group, 13 patients (20%) were HBsAg-positive. No direct evidence that HBV virus represses $p21^{WAF1/CIP1}$ promoter activity has been reported.

Third, proteins can be regulated at the post-transcriptional level. Down-regulation of $p21^{WAF1/CIP1}$ expression through degradation by a ubiquitin-dependent proteolytic pathway has recently been reported (Maki and Howley, 1997). Therefore, we suggest that post-transcriptional regulation may be another mechanism that down-regulates $p21^{WAF1/CIP1}$ expression in HCCs. Drawing together the results of our present and previous (Hui et al, 1997) studies, we consider that the higher incidence of $p21^{WAF1/CIP1}$ protein absence (72.8%) than the incidence of reduced $p21^{WAF1/CIP1}$ mRNA expression (38.1%) further supports this hypothesis. These observations also suggest that analysis of $p21^{WAF1/CIP1}$ status at the protein level may be more sensitive than analysis at the mRNA level.

Fourth, a mutation rate of 5% in $p21^{WAF1/CIP1}$ has been reported in HCCs (Furutani et al, 1997), so mutation of $p21^{WAF1/CIP1}$ may be a possible mechanism leading to altered $p21^{WAF1/CIP1}$ expression in a small proportion of HCCs.

We found concurrent positive expression of $p21^{WAF1/CIP1}$ and p53 in five HCCs. Because p53 overexpression is a hallmark of non-functional p53, the expression of $p21^{WAF1/CIP1}$ in these cases may be induced by p53-independent pathways (Michieli et al, 1994; Zhang et al, 1995).

Two previous studies have evaluated the relationship between the expression of $p21^{WAF1/CIP1}$ and p53 at the protein level in HCCs, although the results were controversial (Qin et al, 1998; Naka et al,

© 2000 Cancer Research Campaign 

1998). Naka et al demonstrated a significant inverse correlation between expression of the two proteins, whereas Qin et al did not. This discrepancy may be due to the difference in hepatitis viral infection status between the two groups of patients, since the molecular basis underlying HCV-related and HBV-related HCCs appears to differ, at least partially. In Qin's group (Chinese patients), the majority (81/97, 84%) of the HCCs were HBV-related, and a small subset (16/97, 16%) were HBsAg-negative. No information about HCV infection was given for this group. On the other hand, in Naka's group (Japanese patients), 38% of HCCs were HBV-related, 44% were HCV-related, and 18% had no hepatitis virus infection. In both of these studies, the relationship between $p21^{WAF1/CIP1}$ and p53 expression in HBV-related HCCs or HCV-related HCCs or HCCs without viral infection was not studied separately. In contrast to the situation in China, the majority (more than 65%) of HCCs in Japan are associated with HCV, and only a small portion (10–15%) are caused by HBV infection (Okuda, 1997). In our study group, 54 of the 81 HCCs (67%) were HCV-related. We found that there was an inverse relationship between $p21^{WAF1/CIP1}$ and p53 expression in HCV-related HCCs, but failed to establish such a relationship in HBV-related HCCs or HCCs without viral infection, suggesting that different modes of $p21^{WAF1/CIP1}$ regulation are involved in HCCs differing in their hepatitis viral infection status. Our data are generally consistent with the results of Qin et al and Naka et al, and offer a credible explanation for the discrepancy between the conclusions of the above two studies.

We found that p53 overexpression was significantly associated with poor tumour differentiation. This is consistent with results of a previous study of p53 in HCCs (Ng et al, 1995). We also found that p53 overexpression was more frequent in tumours with portal vein invasion than in tumours without. These observations suggest that altered p53 may be involved in the progression of HCCs.

Increased $p21^{WAF1/CIP1}$ mRNA levels can make metastatic human melanoma cells lose their metastatic potential (Jiang et al, 1995). We found a tendency for a relatively higher rate of $p21^{WAF1/CIP1}$ expression in the group without intrahepatic metastasis than in the group with intrahepatic metastasis, indicating a potential role of $p21^{WAF1/CIP1}$ dysfunction in the process of HCC metastasis. A correlation between loss of $p21^{WAF1/CIP1}$ expression and tumour metastasis has been previously reported in gastric carcinoma (Ogawa et al, 1997). It is striking that we found no intrahepatic metastasis in 17 HCCs with no abnormality in either the $p21^{WAF1/CIP1}$ or the p53 protein, and that all HCCs with intrahepatic metastasis had altered expression of $p21^{WAF1/CIP1}$, p53, or both. These observations strongly suggest that the p53–$p21^{WAF1/CIP1}$ cell-cycle-regulating pathway may play an important role in suppressing tumour metastasis in HCCs.

We previously showed that other CDK inhibitors, $p16^{INK4}$ (Hui et al, 1996*b*) and $p27^{Kip1}$ (Hui et al, 1998), are involved in hepatocarcinogenesis. $p16^{INK4}$, $p21^{WAF1/CIP1}$ and $p27^{Kip1}$ appear to be independently inactivated and dysfunction of CDK inhibitors is a very common event in HCCs (Hui et al, 1996*a*, 1998; Hui and Makuuchi, 1999). Understanding the mechanisms that inactivate these CDK inhibitors may be important for seeking new biological therapies for HCCs.

In conclusion, our present study has shown that: (1) absence of $p21^{WAF1/CIP1}$ expression is very common in HCCs; (2) $p21^{WAF1/CIP1}$ is probably regulated via different pathways in HCCs differing in their hepatitis viral infection status, and $p21^{WAF1/CIP1}$ expression appears to be predominantly related to altered p53 in HCV-related HCCs; (3) in addition to p53-dependent transcriptional regulation, HCV core protein suppression of the transcriptional activity of the $p21^{WAF1/CIP1}$ promoter and post-transcriptional regulation are alternate pathways by which $p21^{WAF1/CIP1}$ expression can be down-regulated; (4) disruption of the p53–$p21^{WAF1/CIP1}$ cell-cycle-regulating pathway may contribute to malignant progression of HCC.

### ACKNOWLEDGEMENTS

Supported in part by a grant for scientific research (10770607) from the Ministry of Education, Science and Culture of Japan, by a grant from the Kanae Foundation for Life & Socio-Medical Science, Japan, and by a grant from the Public Trust Haraguchi Memorial Cancer Research Fund, Japan (All to A-M Hui).

### REFERENCES

Cote RJ, Dunn MD, Chatterjee SJ, Stein JP, Shi SR, Tran QC, Hu SX, Xu HJ, Groshen S, Taylor CR, Skinner DG and Benedict WF (1998) Elevated and absent pRb expression is associated with bladder cancer progression and has cooperative effects with p53. *Cancer Res* **58**: 1090–1094

Dulic V, Kaufmann WK, Wilson SJ, Tlsty TD, Lees E, Harper JW, Elledge SJ and Reed SI (1994) p53-dependent inhibition of cyclin-dependent kinase activities in human fibroblasts during radiation-induced G1 arrest. *Cell* **76**: 1013–1023

El-Deiry WS, Tokino T, Velculescu VE, Levy DB, Parsons R, Trent JM, Lin D, Mercer WE, Kinzler KW and Vogelstein B (1993) WAF1, a potential mediator of p53 tumor suppression. *Cell* **75**: 817–825

El-Deiry WS, Harper JW, O'Connor PM, Velculescu VE, Canman CE, Jackman J, Pietenpol JA, Burrell M, Hill DE, Wang Y, Wilman KG, Mercer WE, Kastan MB, Kohn KW, Elledge SJ, Kinzler KW and Vogelstein B (1994) WAF1/CIP1 is induced in p53-mediated G1 arrest and apoptosis. *Cancer Res* **54**: 1169–1174

Esrig D, Elmajian D, Groshen S, Freeman JA, Stein JP, Chen S-C, Nichols PW, Skinner DG, Jones PA and Cote RJ (1994) Accumulation of nuclear p53 and tumor progression in bladder cancer. *N Engl J Med* **331**: 1259–1264

Farmer G, Bargonetti J, Zhu H, Friedman P, Prywes R and Prives C (1992) Wild-type p53 activates transcription in vitro. *Nature* **358**: 83–86

Finlay CA, Hinds PW, Tan T-H, Eliyahu D, Oren M and Levine AJ (1988) Activating mutations for transformation by p53 produce a gene product that forms an hsc70–p53 complex with an altered half-life. *Mol Cell Biol* **8**: 531–539

Furutani M, Arii S, Tanaka H, Mise M, Niwano M, Harada T, Higashitsuji H, Imamura M and Fujita J (1997) Decreased expression and rare somatic mutation of the CIP1/WAF1 gene in human hepatocellular carcinoma. *Cancer Lett* **111**: 191–197

Gu Y, Turck CW and Morgan DO (1993) Inhibition of CDK2 activity in vivo by an associated 20K regulatory subunit. *Nature* **366**: 707–710

Hall PA, Ray A, Lemoine NR, Midgley CA, Krausz T and Lane DP (1991) p53 immunostaining as a marker of malignant disease in diagnostic cytopathology. *Lancet* **338**: 513

Harper JW, Adami GR, Wei N, Keyomarsi K and Elledge SJ (1993) The p21 Cdk-interacting protein Cip1 is a potent inhibitor of G1 cyclin-dependent kinases. *Cell* **75**: 805–816

Hui A-M and Makuuchi M (1999) Molecular basis of multistep hepatocarcinogenesis: genetic and epigenetic events. *Scand. J Gastroenterol* **34**: 737–742

Hui A-M, Sakamoto M, Kanai Y, Ino Y, Gotoh M and Hirohasi S (1996*a*) Cyclin-dependent kinase inhibitors and hepatocarcinogenesis. In: *Recent Advances in Gastroenterological Carcinogenesis*, Tahara E, Sugimachi K and Oohara T (eds), pp. 481–485. Monduzzi Editore, Bologna

Hui A-M, Sakamoto M, Kanai Y, Ino Y, Gotoh M, Yokota J and Hirohashi S (1996*b*) Inactivation of $p16^{INK4}$ in hepatocellular carcinoma. *Hepatology* **24**: 575–579

Hui A-M, Kanai Y, Sakamoto M, Tsuda H and Hirohashi S (1997) Reduced $p21^{WAF1/CIP1}$ expression and p53 mutation in hepatocellular carcinomas. *Hepatology* **25**: 575–579

Hui A-M, Makuuchi M and Li X (1998*a*) Cell cycle regulators and human hepatocarcinogenesis. *Hepato-Gastroenterology* **45**: 1635–1642

Hui A-M, Sun L, Kanai Y, Sakamoto M and Hirohashi S (1998*b*) Reduced $p27^{kip1}$ expression in hepatocellular carcinomas. *Cancer Lett* **132**: 67–73

Hui A-M, Cui X, Makuuchi M, Li X, Shi Y-Z and Takayama T (1999*a*) Decreased $p27^{Kip1}$ expression and cyclin D1 overexpression, alone and in combination,

© 2000 Cancer Research Campaign influence recurrence and survival of patients with resectable extrahepatic bile duct carcinoma. *Hepatology* **30**: 1167–1173

Hui A-M, Li X, Makuuchi M, Takayama T and Kubota K (1999*b*) Over-expression and lack of retinoblastoma protein are associated with tumor progression and metastasis in hepatocellular carcinoma. *Int J Cancer* **84**: 604–608

Hui A-M, Shi Y-Z, Li X, Takayama T and Makuuchi M (2000) Loss of p16$^{INK4}$ protein, alone and together with loss of retinoblastoma protein, correlate with hepatocellular carcinoma progression. *Cancer Lett* in press.

Hunter T and Pines J (1994) Cyclins and cancer II: cyclin D and CDK inhibitors come of age. *Cell* **79**: 573–582

Iggo R, Gatter K, Bartek J, Lane D and Harris AL (1990) Increased expression of mutant forms of p53 oncogene in primary lung cancer. *Lancet* **335**: 675–679

Jiang H, Lin J, Su ZZ, Herlyn M, Kerbel RS, Weissman BE, Welch DR and Fisher PB (1995) The melanoma differentiation-associated gene mda-6, which encodes the cyclin-dependent kinase inhibitor p21, is differentially expressed during growth, differentiation and progression in human melanoma cells. *Oncogene* **10**: 1855–1864

Li X, Hui A-M, Takayama T, Cui X, Shi Y-Z and Makuuchi M (2000) Altered p21$^{WAF1/CIP1}$ expression is associated with poor prognosis in extrahepatic bile duct carcinoma. *Cancer Lett* (in press)

Loda M, Cukor B, Tam SW, Lavin P, Fiorentino M, Draetta GF, Jessup JM and Pagano M (1990) Increased proteasome-dependent degradation of the cyclin-dependent kinase inhibitor p27 in aggressive colorectal carcinomas. *Nat Med* **3**: 231–234

Maki CG and Howley PM (1997) Ubiquitination of p53 and p21 is differentially affected by ionizing and UV radiation. *Mol Cell Biol* **17**: 355–363

Michieli P, Chedid M, Lin D, Pierce JH, Mercer WE and Givol D (1994) Induction of WAF1/CIP1 by a p53-independent pathway. *Cancer Res* **54**: 3391–3395

Naka T, Toyota N, Kaneko T and Kaibara N (1998) Protein expression of p53, p21$^{WAF1}$, and Rb as prognostic indicators in patients with surgically treated hepatocellular carcinoma. *Anticancer Res* **18**: 555–564

Ng IO, Lai EC, Chan AS and So MK (1995) Overexpression of p53 in hepatocellular carcinomas: a clinicopathological and prognostic correlation. *J Gastroenterol Hepatol* **10**: 250–255

Noda A, Ning Y, Venable SF, Pereira-Smith OM and Smith JR (1994) Cloning of senescent cell-derived inhibitors of DNA synthesis using an expression screen. *Exp Cell Res* **211**: 90–98

Ogawa M, Maeda K, Onoda N, Chung YS and Sowa M (1997) Loss of p21$^{WAF1/CIP1}$ expression correlates with disease progression in gastric carcinoma. *Br J Cancer* **75**: 1617–1620

Okuda K (1997) Hepatitis C virus and hepatocellular carcinomas. In: *Liver Cancer*, Okuda K and Tabor E (eds), pp. 39–50. Churchill Livingstone Edinburgh

Qin LF, Ng IO, Fan ST and Ng M (1998) p21/WAF1, p53 and PCNA expression and p53 mutation status in hepatocellular carcinoma. *Int J Cancer* **79**: 424–428

Ray RB, Steele R, Meyer K and Ray R (1998) Hepatitis C virus core protein represses p21$^{WAF1/CIP1/Sid1}$ promoter activity. *Gene* **208**: 331–336

Sarnow P, Ho YS, Williams J and Levine AJ (1982) Adenovirus E1b-58kd tumor antigen and SV40 large tumor antigen are physically associated with the same 54 kDa cellular protein in transformed cells. *Cell* **28**: 387–394

Sheikh MS, Rochefort H and Garcia M (1995) Overexpression of p21$^{WAF1/CIP1}$ induces growth arrest, giant cell formation and apoptosis in human breast carcinoma cell lines. *Oncogene* **11**: 1899–1905

Somasundaram K and El-Deiry WS (1997) Inhibition of p53-mediated transactivation and cell cycle arrest by E1A through its p300/CBP-interacting region. *Oncogene* **14**: 1047–1057

Steegenga WT, van Laar T, Riteco N, Mandarino A, Shvarts A, van der Eb AJ and Jochemsen AG (1996) Adenovirus E1A proteins inhibit activation of transcription by p53. *Mol Cell Biol* **16**: 2101–2109

Waga S, Hannon GJ, Beach D and Stillman B (1994) The p21 inhibitor of cyclin-dependent kinases controls DNA replication by interaction with PCNA. *Nature* **369**: 574–578

Xiong Y, Hannon GJ, Zhang H, Casso D, Kobayashi R and Beach D (1993) p21 is a universal inhibitor of cyclin kinases. *Nature* **366**: 701–704

Yew PR and Berk AJ (1992) Inhibition of p53 transactivation required for transformation by adenovirus early 1B protein. *Nature* **357**: 82–85

Zhang W, Grasso L, McClain CD, Gambel AM, Cha Y, Travali S, Deisseroth AB and Mercer WE (1995) p53-independent induction of WAF1/CIP1 in human leukemia cells is correlated with growth arrest accompanying monocyte/macrophag differentiation. *Cancer Res* **55**: 668–674

© 2000 Cancer Research Campaign 
